# Adult pneumococcal meningitis presenting with normocellular cerebrospinal fluid: two case reports

**DOI:** 10.1186/1752-1947-7-294

**Published:** 2013-12-31

**Authors:** Hiromichi Suzuki, Yasuharu Tokuda, Yoko Kurihara, Masatsune Suzuki, Hidenori Nakamura

**Affiliations:** 1 Department of Clinical Laboratory Medicine, Tsukuba Medical Center Hospital, 1-3-1 Amakubo, Tsukuba 305-8558, Japan; 2 Department of Medicine, Mito Kyodo General Hospital, University of Tsukuba, 3-2-7 Miyamachi, Mito 310-0015, Japan; 3 Department of Infectious Diseases, University of Tsukuba Hospital, 2-1-1 Amakubo, Tsukuba 305-8576, Japan; 4 Department of General Medicine and Primary Care, Tsukuba Medical Center Hospital, 1-3-1 Amakubo, Tsukuba 305-8558, Japan; 5 Department of Respiratory Medicine, Seirei Hamamatsu General Hospital, 2-12-12 Sumiyoshi, Naka-ku, Hamamatsu 430-8558, Japan

**Keywords:** Cerebrospinal fluid, Diagnosis, Pneumococcal meningitis, *Streptococcus pneumoniae*

## Abstract

**Introduction:**

Normocellular bacterial meningitis is rarely observed in adult patients. We here report two cases of adult patients with pneumococcal meningitis with a normal cerebrospinal fluid leukocyte count and review eight other cases in the literature.

**Case presentation:**

Case 1 was a 34-year-old Japanese woman with a history of splenectomy who presented with pyrexia, nausea, headache, and loss of hearing in her right ear. She was in a hypotensive state with no neck stiffness and had a normal mental status at the initial presentation. She became progressively disoriented during out-patient management. A cerebrospinal fluid examination showed a normal leukocyte count despite the presence of *Streptococcus pneumoniae*, which was detectable with Gram staining. She survived after prompt treatment, but her hearing loss remained. Case 2 was a 62-year-old Japanese man with a history of laryngeal cancer who was transferred to our emergency department after an acute onset of delirium and rapid progression to septic shock. As in Case 1, cerebrospinal fluid examination showed a normal leukocyte count despite the presence of *S. pneumoniae*, which was detectable with Gram staining. Within 1 hour of arrival, he developed hypotension and subsequent cardiopulmonary arrest, and resuscitation was unsuccessful.

**Conclusions:**

These cases imply that a normal leukocyte count in the cerebrospinal fluid does not exclude the possibility of bacterial meningitis. Gram staining of cerebrospinal fluid and immediate administration of antibiotics should be performed in all patients with suspected bacterial meningitis.

## Introduction

Lumbar puncture and examination of the cerebrospinal fluid (CSF) are essential steps in the diagnosis of meningitis. Typically, the CSF shows significant neutrophilic pleocytosis [1], a decreased glucose concentration, and an elevated protein level. The absence of pleocytosis in the CSF has occasionally been described in cases of pediatric meningitis [2,3], especially in the very acute phase of bacterial meningitis [2]. By contrast, bacterial meningitis with a normal CSF leukocyte count has rarely been described in adults. We herein describe cases involving two adult patients with normocellular pneumococcal meningitis and present a review of eight previous cases.

## Case presentation

### Case 1

A 34-year-old Japanese woman presented to our hospital one morning because of nausea, headache, and loss of hearing in her right ear, all of which had developed a few hours before visiting our hospital. She also stated that she had a fever of 39.7°C, which had suddenly developed after suffering chills two nights previously. She had a history of splenectomy as a result of a traffic accident, but had not received a pneumococcal vaccine. She had received oral cephalosporin (cefditoren pivoxil) and an antipyretic analgesic (loxoprofen sodium hydrate), which were prescribed at a nearby clinic, for the fever on the day before her hospital visit.

On initial physical examination, she was afebrile (36.6°C), but in a hypotensive state (88/55mmHg) with tachycardia (120 beats/minute). There was no obvious deficiency in her mental status nor was neck stiffness detected at initial presentation, but she became progressively disoriented (Glasgow coma scale rating of E3V4M6) during out-patient management. She was admitted to the intensive care unit and treated with fluid replacement and a vasopressor. Blood tests showed an elevated leukocyte count (27,400/μL) with a left shift, elevated C-reactive protein level (27.3mg/dL), and low platelet count (89,000/μL). Examination of a CSF sample obtained 90 minutes after presenting to the hospital demonstrated a normal cell count (2/μL) without red blood cells. The CSF was clear, and the concentrations of glucose and total protein in the CSF were 56mg/dL (blood glucose level: 92mg/dL) and 40mg/dL, respectively (Table 1). Gram staining of the CSF showed Gram-positive cocci that were subsequently identified in culture as *Streptococcus pneumoniae*. Penicillin G was initially administered in the emergency department, and combination therapy with cefotaxime and vancomycin was started after admission. CSF examination on day 2 of hospitalization showed marked pleocytosis (mononuclear cells: 2448/μL, polymorphonuclear cells: 1638/μL), an increased total protein level (270mg/dL), and a decreased glucose level (13mg/dL). Two blood samples for culture were obtained simultaneously with the start of intravenous antimicrobial therapy in the emergency department, but no bacteria were cultivated. The drug susceptibilities of *S. pneumoniae* grown in the CSF cultures were determined by the disk dilution method and showed susceptibility to penicillin (≥20mm), erythromycin (≥21mm), and levofloxacin (≥17mm). She recovered after a 3-week treatment with these antimicrobial agents and was discharged on day 23. Her hearing loss remained.

**Table 1:** Results of cerebrospinal fluid examination (Case 1)

	**Day 1**	**Day 2**	**Day 14**
Cell/PMN(/μL)	2/1	4086/1638	13/2
Glucose (mg/dL) (blood glucose)	56 (92)	13 (127)	52 (123)
Protein (mg/dL)	40	270	26
Gram stain	GPC	Not performed	Negative

*GPC* Gram-positive cocci, *PMN* Polymorphonuclear leukocytes.

### Case 2

A 62-year-old Japanese man with a history of treatment for laryngeal cancer 7 years previously was transferred to the emergency department after an acute onset of delirium. He had developed a fever the previous day. He did not receive antimicrobial therapy before being transferred. Neither neck stiffness nor paralysis was apparent on physical examination. Blood tests revealed a low leukocyte count (2480/μL), elevated C-reactive protein level (33.0mg/dL), and low platelet count (38,000/μL). CSF examination demonstrated a normal cell count (1/μL) without red blood cells. A low CSF glucose level of 8mg/dL (blood glucose level: 69mg/dL) and a high total protein level of 125mg/dL were present. Gram staining of the CSF showed numerous Gram-positive cocci that were proven to be *S. pneumoniae* by CSF culture. Blood cultures were not obtained. A chest X-ray showed an infiltrate in his left lower lung field. Within 1 hour of arrival, he developed hypotension followed by cardiopulmonary arrest, and resuscitation was unsuccessful. The drug susceptibilities of *S. pneumoniae* were determined by the microdilution method with MicroScan WalkAway^®^ (Siemens Healthcare) and showed susceptibility to ampicillin (≤0.5μg/mL), cefotaxime (≤0.5μg/mL), and meropenem (≤1μg/mL), but resistance to penicillin (0.12μg/mL) and erythromycin (≥2μg/mL).

## Discussion

In both cases in this report, CSF examinations showed normal leukocyte counts despite the presence of *S. pneumoniae*, which was detectable with Gram staining. Both patients had an acute onset of fever accompanied by neuropsychological signs and symptoms and rapid progression to septic shock. Clinical evaluations were performed immediately after hospital presentation in both cases, and lumbar puncture was performed less than half a day after neurological symptoms appeared in Case 1. The findings in these cases indicate that the CSF can appear normal, especially when a lumbar puncture is performed soon after the start of symptoms. The first patient survived with hearing loss after prompt treatment, but the second patient died prior to administration of antibiotics.

To the best of our knowledge, only eight cases of adult patients with pneumococcal meningitis with a normal CSF cell count have been previously reported [4-9]. The clinical characteristics of the previous and current cases are summarized in Table 2. All patients with the exception of one were younger than 65 years, and seven were male. Most patients did not have significant comorbidities, but one had alcoholism, one had Hodgkin’s disease, and one had undergone splenectomy (present Case 1). Pneumonia was present in four patients as another infection site, and the mortality rate was 40% (4 out of 10).

**Table 2:** Summary of reported cases of adult pneumococcal meningitis with normal cerebrospinal fluid cell counts

**Case/authors**	**Age/sex** **(years)**	**Underlying condition**	**Other site of infection**	**Cerebrospinal fluid examination**				**Antibiotics**	**Outcome**
				**Cell/PMN (/μL)**	**Glucose (mg/dL)**	**Protein (mg/dL)**	**Gram stain**		
1/Fishbein *et al.*[4]	33/M	Hodgkin’s disease	None	0/0	1	685	GPC	CET→CP	Death
2/Fishbein *et al.*[4]	57/M	Alcoholism	Pneumonia	1/0	130	97	Negative	PCG	Survival
3/Fishbein *et al.*[4]	86/M	None	Pneumonia	0/0	221	40	GPC	PCG	Death
4/Ris *et al.*[5]	50/F	None	Pneumonia	0/0	95	21	Negative	CTX→PCG	Survival
5/Zenebe [6]	18/M	ND	None	0/0	4	300	GPC	None	Death
6/Uchihara *et al.*[7]	52/M	None	None	6/2	71	26	ND	CMX→PCG→ABPC+RFP	Survival
7/Montassier *et al.*[8]	60/F	None	None	0/0	70	39	Negative	CTRX	Survival
8/Alvarez *et al.*[9]	37/M	None	None	1/ND	52	47	Negative	VCM+CFPM+MNZ→CTRX	Survival
9/present case 1	34/F	Splenectomy	None	2/1	56	40	GPC	CTX+VCM→CTX	Survival
10/present case 2	62/M	None	Pneumonia	1/0	8	125	GPC	None	Death

*ABPC* Ampicillin, *CET* Cephalothin, *CFPM* Cefepime, *CMX* Cefmenoxime, *CP* Chloramphenicol, *CTRX* Ceftriaxone, *CTX* Cefotaxime, *F* Female, *GPC* Gram-positive cocci, *M* Male, *MNZ* Metronidazole, *ND* Not described, *PCG* Penicillin G, *PMN* Polymorphonuclear leukocytes, *RFP* Rifampin, *VCM* Vancomycin.

In adult patients with bacterial meningitis, a low white blood cell count in the CSF is an important risk factor for a poor prognosis [10]. Weisfelt *et al.* found that a CSF leukocyte count of <1000/μL was correlated with morbidity and mortality in patients with pneumococcal meningitis [11] and suggested that a low CSF leukocyte count might reflect inadequate host defense against bacterial infections in adult patients with bacterial meningitis. Case 1 showed increased pleocytosis in the second CSF analysis, which indicated a delayed immunological response due to the lack of spleen function.

In addition to a low CSF cell count, a positive CSF Gram stain result, indicating a high number of *S. pneumoniae* microorganisms, is also a poor prognostic factor in patients with bacterial meningitis [12]. Of the 10 patients (Table 2), four had negative findings on the initial Gram stains, and all of these patients survived. By contrast, four of five patients with Gram-positive cocci observed on the initial Gram stains died after rapid progression of the clinical course [4,6], and the only survivor (Case 1 in this report) had persistent hearing loss.

## Conclusions

The present and previous cases suggest that bacterial meningitis should be considered in adult patients suspected to have bacterial meningitis, even if the initial CSF cell count is normal, and that Gram staining of the CSF and prompt administration of an adequate dose of antibiotics should be performed with a second CSF examination the following day. This approach is important because the CSF cell count may not be elevated in the first CSF examination, and the presence of Gram-positive cocci in the CSF is associated with high mortality and morbidity in patients with normocellular pneumococcal meningitis.

## Consent

Written informed consent was obtained from the patient described in Case 1 for publication of this case report, and a copy of the written consent is available for review by the Editor-in-Chief of this journal. Although the patient described in Case 2 died of pneumococcal meningitis, the hospital ethics committee confirmed that written informed consent could be waived because the family agreed to the anonymous use of the patient’s data for clinical research purposes. A copy of the certification of approval is available for review by the Editor-in-Chief of this journal.

## Competing interests

The authors declare that they have no competing interests.

## Authors’ contributions

YK and MS were involved in the clinical diagnostic evaluation and management. HS, HN, and YT analyzed and interpreted the patient data and were major contributors in writing the first draft. All authors reviewed and approved the final manuscript.
